# The Toxicity Mechanisms of Action of Aβ_25–35_ in Isolated Rat Cardiac Myocytes

**DOI:** 10.3390/molecules190812242

**Published:** 2014-08-13

**Authors:** Beiru Zhang, Xiaohui Bian, Ping He, Xiaoying Fu, Keiichi Higuchi, Xu Yang, Detian Li

**Affiliations:** 1 Department of Nephrology, Shengjing Hospital, China Medical University, Shenyang 110004, China; E-Mails: bxh_xh@163.com (X.B.); hping0711@sohu.com (P.H.); yx-xu@outlook.com (X.Y.); lidt_sjh@163.com (D.L.); 2 Department of Pathology, Tianjin University of Traditional Chinese Medicine, Tianjin 300193, China; E-Mail: fu_xiao_y@126.com; 3 Department of Aging Biology, Institute of Pathogenesis and Disease Prevention, Shinshu University Graduate School of Medicine, Shinshu University, 3-1-1, Asahi, Matsumoto 390-8621, Japan; E-Mail: keiichih@shinshu-u.ac.jp

**Keywords:** Alzheimer’s disease (AD), β-amyloid (Aβ), rat cardiac myocyte, ER-stess, p38

## Abstract

β-Amyloid (Aβ) is deposited in neurons and vascular cells of the brain and is characterized as a pathologic feature of Alzheimer’s disease (AD). Recently studies have reported that there is an association between cardiovascular risk factors and AD, however the mechanism of this association is still uncertain. In this study we observed Aβ had an effect on cardiovascular cells. We represent as a major discovery that Aβ_25–35_ had toxicity on isolated rat cardiac myocytes by impacting the cytoskeleton assembly and causing ER stress, ultimately contributing to the apoptosis of the myocytes. Importantly, the activation of ER stress and subsequent cellular dysfunction and apoptosis by Aβ_25–35_ was regulated by the MAPK pathway, which could be prevented by inhibition of p38 via pharmacological inhibitors. It was noteworthy that Aβ_25–35_ played a critical role in cardiac myocytes, suggesting that Alzheimer’s disease (AD) had a relation with the heart and understanding of these associations in future will help search for effective treatment strategies.

## 1. Introduction

Alzheimer’s disease (AD) is the most common neurodegenerative disease all over the world, representing more than 60% of all cases. It is estimated that approximately 35 million people are being affected by AD worldwide, and the number of affected individuals is expected to grow dramatically [1]. The two major pathologic features of AD are extracellular amyloid beta (Aβ) plaques and intracellular neurofibrillary tangles (NFT). They are abnormally folded and accumulated in the brains of AD patients [2]. The mutation in the genes of amyloid precursor protein (APP) and presenilin (PS) contribute to the excessive amount of misfolded amyloid peptides and the mutation in the gene of apolipoprotein E (ApoE) leads to altered clearance and transport of Aβ, resulting in the amyloid plaque deposit [3].

Among the amyloid plaques, Aβ as a full length peptide with 40–43 amino acids, is the critical component. Aβ comes from the amyloidogenic processing of the β-amyloid precursor protein (APP). APP is a kind of ubiquitously single-pass transmembrane protein, that is sequentially cleaved by the sequential action of β-secretase/BACE1 and γ-secretase [4]. However, the interesting property of Aβ peptide is that there are various fragments including residues 1–28 [5], 25–35 [6], and 34–42 [7], and they also show similar biophysical and biochemical properties as full-length Aβ peptide [8].

Recently many evidences suggest that there is close relationship between Alzheimer’s disease and cardiovascular disease, especially cardiac insufficiency. Compared to the wild rat, the APPswe/PS1dE9 mouse with Alzheimer’s disease has obvious cardiomyocyte contractile dysfunction, suggesting that Aβ maybe affects the mouse cardiomyocytes and leads to the cardiomyocyte contractile dysfunction [9]. Intracellular accumulation of β-amyloid were seen by using ultrastructural tests in a cardiac biopsy taken from a heart with amyloidosis, suggesting that heart issue may be another organ where amyloidogenic peptide leads to cardiomycocyte destruction and heart dysfunction [10,11]. Liao reported in 2009 that amyloidogenic light chain (AL-LC) proteins provoked oxidative stress, cellular dysfunction, and apoptosis in isolated adult cardiomyocytes [12]. At present the mechanism whereby β-amyloid (Aβ) could damage the organism is still unclear. However, it has been reported that human amyloidogenic precursor proteins directly impair cell function without forming amyloid fibrils.

Based on the above study we demonstrated for the first time that Aβ_25–35_ as one of the active fragments of Aβ could have an impact on rat cardiac myocytes *in vitro*. Using isolated rat cardiac myocytes, we proved Aβ_25–35_ directly caused cardiac myocyte ER stress and cytoskeletal changes, ultimately leading to apoptosis. These observations agreed with the report that Aβ peptide induced neuronal ER stress leading to the activation of the mitochondrial apoptotic pathway. It has been reported that mitogen-activated protein kinase (MAPK) could be activated in AD disease [13]. We, therefore, set out to determine whether MAPK could be a key mediator involved in the regulation of Aβ induced apoptosis and the ER stress on rat cardiac myocytes *in vitro*.

Herein, using cardiac myocyte isolated from rat, we demonstrated that Erk1/2 and p38 MAPK is immediately and differentially regulated by Aβ_25–35_. Importantly, inhibition of the p38 pathway by its selective inhibitor SB203580 prevented Aβ-induced cardiac myocyte apoptosis, suggesting that activation of p38 through ER stress may plays a pivotal role in triggering Aβ-induced cellular dysfunction and potentially leads to the subsequent pathogenesis of cardiac amyloidosis.

## 2. Results

### 2.1. Effect of Aβ_25–35_ on the Viability of Rat Cardiac Myocyte in Vitro

**Figure 1:**
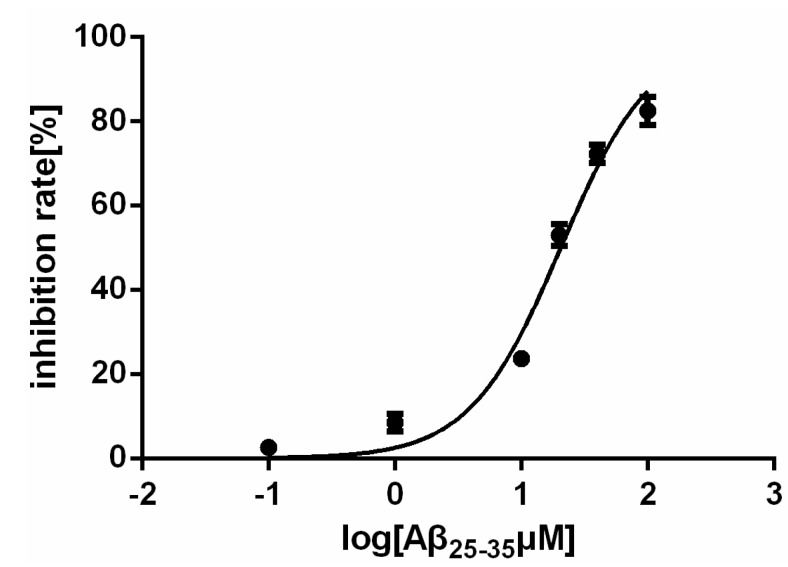
The viability of the myocardial cells is affected by Aβ_25–35_.

The Aβ_25–35_ fragment of the Alzheimer amyloid β-peptide, like its full-length peptide Aβ (1–42), has shown toxic activities in cultured cells [8,14]. The effect of Aβ (1–42) on cultured neurons is concentration-dependent, and significant cell loss was detected after treatment at concentrations of 20 μM. Although these Aβ concentrations are higher those seen in pathological conditions, low plasma Aβ levels could still cause a big negative impact due to extended stimulation [14,15,16,17]. We studied whether the fragment Aβ_25–35_ has the ability to inhibit the growth of rat cardiac myocytes. The cell growth-inhibitory activity was determined by colorimetric measurement of cell viability. Firstly the rat cardiac mycocytes were cultured overnight and then treated with different concentrations of Aβ_25–35_ for another 24 h. The results show that Aβ_25–35_ caused a progressive increase in apoptotic cell death and the IC_50_ is 20.52 μM (Figure 1). This result indicated Aβ_25–35_ could inhibit the viability of cardiac myocytes. Higher concentrations of Aβ_25–35_ result in cell death and secondary apoptosis. Thus, a concentration range of 10–40 μM Aβ_25–35_ was chosen in this study for the exploration of the influence of Aβ_25–35_ on rat cardiac mycocytes without having a lethal impact.

### 2.2. Aβ_25–35_ Induced Cardiac Myocytes Apoptosisfigure

**Figure 2:**
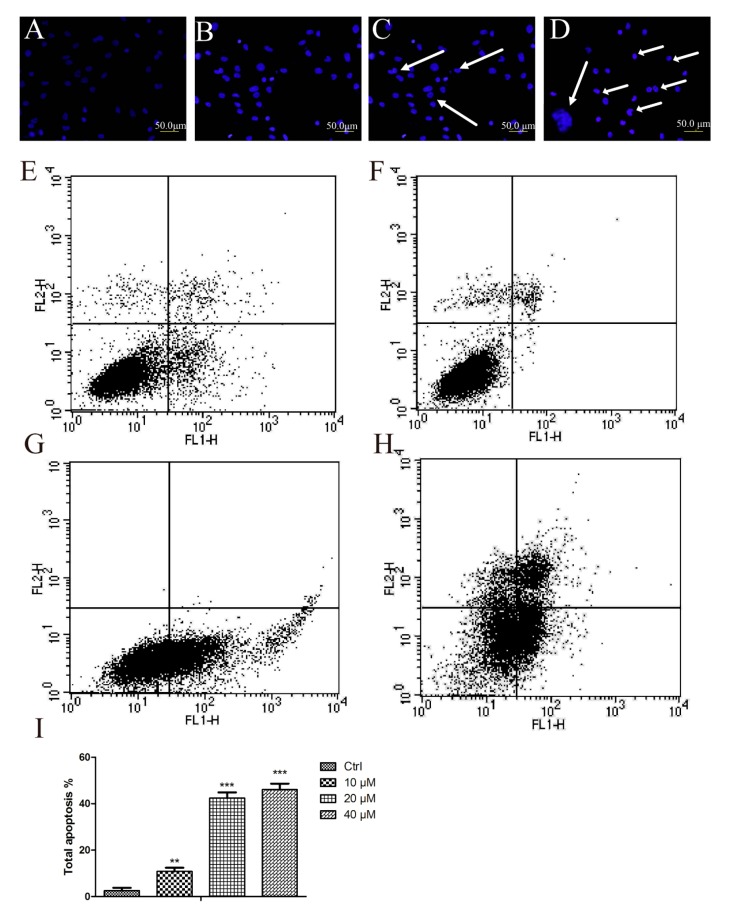
Aβ_25–35_ induced cardiac myocytes apoptosis. (**A**–**D**) Morphologic changes of the cardiac myocyte nuclei induced by Aβ_25–35_. Cultured cardiac myocyte were treated with Aβ_25–35_ at the indicated concentrations for 24 h. The cells were stained with Hoechst and observed by fluorescence microscopy; (**E**–**H**) Aβ_25–35_ induces apoptosis in a dose-dependent way. Cardiac myocyte were treated with different concentrations of Aβ_25–35_ for 24 h, and then stained with propidium iodide for flow cytometry analyses; (**A**–**E**), control (**B**–**F**), 10 μM (**C**–**G**), 20 μM (**D**–**H**), 40 μM; (**I**), The apoptosis rate induced by Aβ_25–35_. Values are expressed as mean ± SEM of three independent experiments, each in triplicate. ** *p* < 0.01, *** *p* < 0.001 *vs.* the control group.

**Figure 3:**
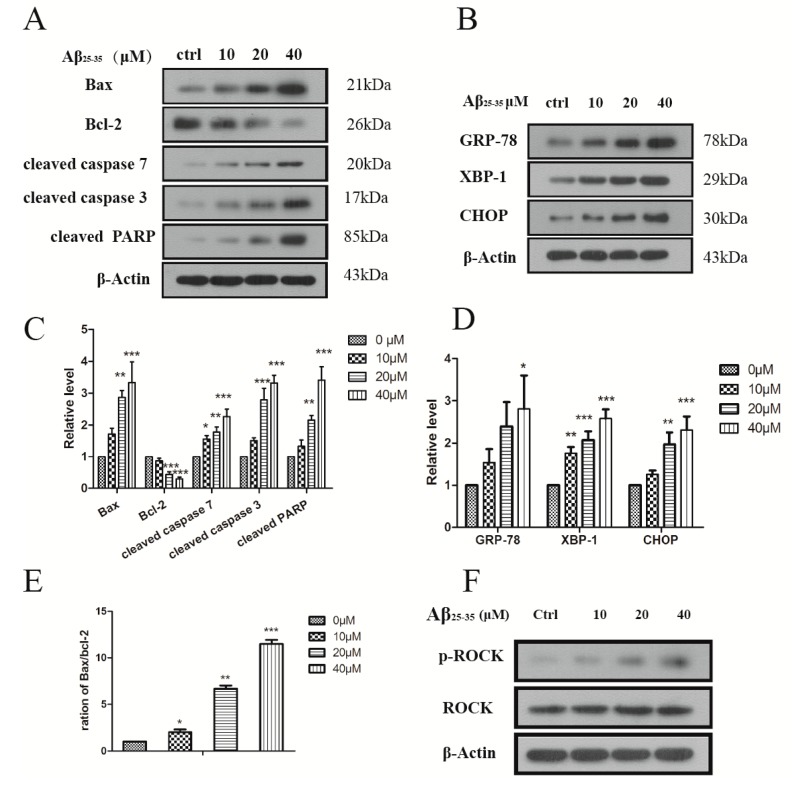
(**A**) Aβ_25–35_ induced the expression of the apoptosis protein in the cardiac myocyte; (**B**) Aβ_25–35_ induced the expression of ER stress marker in the cardiac myocyte. Cells were treated with indicated concentrations of Aβ_25–35_ for 24 h and subjected to western blotting to measure the related protein; (**C**–**D**) Relative protein levels were quantified by densitometry and shown in the histogram; (**E**) The ratio of the Bax/Bcl-2; (**F**) Aβ_25–35_ decreased the expression ROCK protein. Values are expressed as mean ± SEM of three independent experiments, each in triplicate. * *p* < 0.05, ** *p* < 0.01, *** *p* < 0.001 *vs.* the control group.

Aβ_25–35_ affected the viability of cardiac mycocytes. In order to detect Aβ_25–35_-mediated apoptosis in myocytes, the Hoechst and Annexin V/PI staining methods were applied. In Hoechst staining, control cells emitted a blue fluorescence with consistent nucleus intensity and presented a typical homogeneous distribution of chromatin in the nucleus. In contrast, cells treated with Aβ_25–35_ for 24 h presented the morphological features of early apoptotic cells, especially apoptotic bodies and nuclei pyknosis. These features appeared more frequently with increasing concentrations. To further study the effect of Aβ_25–35_ on cardiac myocytes, a quantitative analysis of apoptotic cells was performed by flow cytometry. As Figure 2E–H show, Aβ_25–35_ treatment resulted in a significant, dose-dependent induction of apoptosis compared with control. Treatments with 40 μM Aβ_25–35_ for 24 h induced more than 46.1% of cells to total apoptosis and more than 15.68% of cells to early apoptosis (Figure 2I). In order to corroborate these results, we determined some well-established biochemical markers of apoptosis by immunoblotting. Indeed, Aβ_25–35_ increased the levels of cleaved caspase 3, 7/PARP in a concentration dependent fashion (Figure 3A,C). The expression of Bax (an apoptosis promoter) was dramatically higher and Bcl-2 (an apoptosis inhibitor) was significantly decreased compared with control in the cardiac mycocytes. It was confirmed that Aβ_25–35_ could induced cardiac myocyte apoptosis by an enhanced Bax/Bcl-2 signal pathway and showed toxicity on these normal cells.

### 2.3. Aβ_25–35_ Induced ER Stress in Cardiac Myocytes Cell

Previously it was reported that Aβ could lead to endoplasmic reticulum stress in cultured cortical neurons [18]. Now we further sought to detect by western blot (WB) whether Aβ_25–35_ would induce the ER stress in cardiac myocytes by measuring the protein levels of ER stress markers. XBP-1 is a key mediator of the ER unfolded protein response (UPR), and GRP78 is the chaperone [19]. Results showed that after treatment with different concentrations of Aβ_25–35_ the levels of XBP and Grp78 were significantly increased and the effect started at 10 μM after 24 h incubation. Prolonged ER stress would promote the up-regulation of a transcription factor C/EBP homologous protein (CHOP), which down-regulates the level of anti-apoptotic protein Bcl-2, further leading to apoptosis [20]. Indeed, in our results the ER stress induced by Aβ_25–35_ could contribute to the high level of CHOP, decreasing the Bcl-2 protein as seen in Figure 3B. These results suggested that Aβ_25–35_ led to ER stress, which in turn reduced Bcl-2 activation in the cardiac myocytes.

### 2.4. Aβ_25–35_ Affects the Cytoskeleton Assembly in Cardiac Myocyte Cells

**Figure 4:**
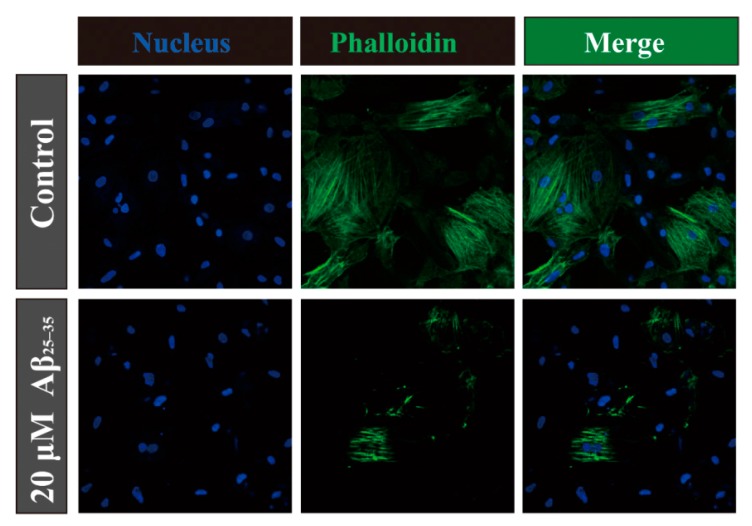
Staining of actin filaments in cardiac myocyte with phalloidin tagged with Alexa Fluor 488 (panel in the mid column) after 24 h of incubation. Panel in the right column represents their corresponding merge images with DAPI. Arrow-heads (in white) indicate damaged actin filaments.

Microtubules and actin filaments play important roles in mitosis, cell signaling and cell-motility. The amyloid precursor protein (APP) is involved in the pathogenesis of Alzheimer’s disease, and the amyloid precursor protein intracellular domain (AICD) could disrupt actin dynamics and mitochondrial bioenergetics [21]. However, Aβ_25–35_ is the cleaved product from APP. In order to confirm the role Aβ_25‑35_ plays in cardiac myocytes, we applied immunofluorescence assays for the determination of cytoskeletal assembly. When the cardiac myocytes were treated with Aβ_25–35_ for 24 h, actin filaments became dramatically destabilized compared with the control (Figure 4). In addition ROCKs control actin-cytoskeleton assembly and cell contractility, and thereby contribute to several physiological processes [22]. We also detected the level of ROCKs protein on the cardiac myocyte cells. As Figure 3F shows Aβ_25–35_ increased the expression of phosphorylated ROCK protein (phospho T249) in a concentration dependent way without any effect on the total protein. Evidence suggested that the damaged cell’s cytoskeleton could contribute cell with the poor viability and even apoptosis fates. It is possible that there is a relationship between Aβ_25–35_-induced cytoskeleton assembly activity and ROCK protein, however further study is needed to conform this.

### 2.5. Activation of p38 and ERK1/2 by Aβ_25–35_ in Cardiac Myocytes Cell

**Figure 5:**
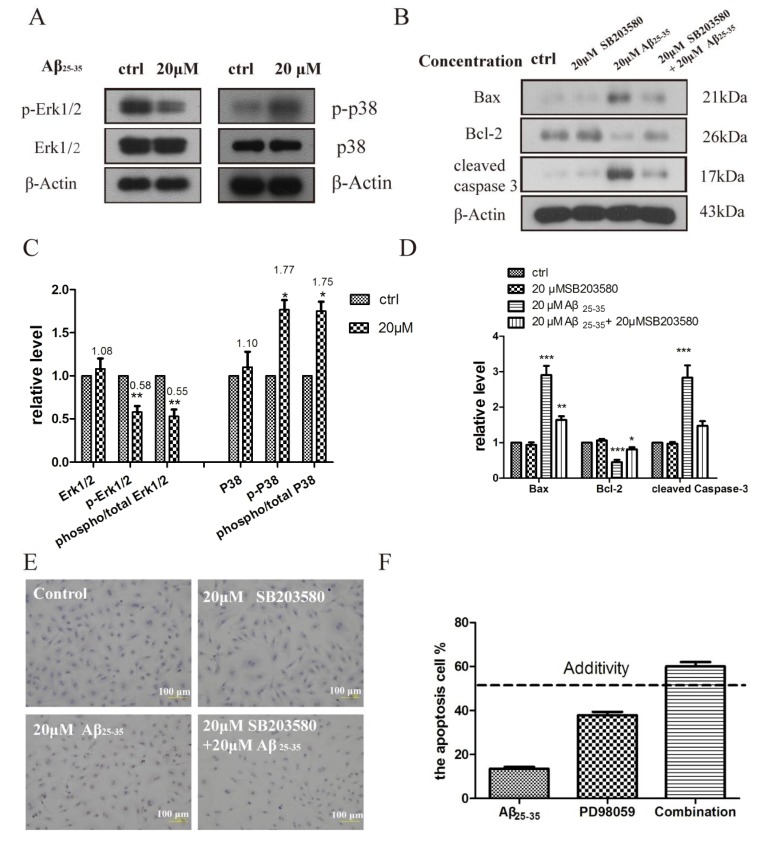
(**A**) Aβ_25–35_ regulates MAPK in isolated rat cardiomyocytes. Immunoblots showing phospho- and total p38, and ERK MAPKs in isolated cardiomyocytes incubated with vehicle, Aβ_25–35_ (20 μM) for 24 h; (**B**) Aβ_25–35_ induced apoptosis protein was inhibited in cardiomyocytes by SB203580 (20 μM) that was added 30 min before the addition of vehicle and Aβ_25–35_; (**C**–**D**) Relative protein levels were quantified by densitometry and shown in the histogram; (**E**) Aβ_25–35_ and PD98059 synergistically induced the cardiac myocyte apoptosis. The cardiac myocyte were treated for 24 h with 10 μM Aβ_25–35_ and 20 μM PD98059. The apoptosis rates were determined by flow cytometry analyses; (**F**) Aβ_25–35_ induced TUNEL-positive apoptotic cells was inhibited in cardiomyocytes by SB203580 (20 μM). TUNEL assay was performed, as described in material and methods section, cardiomyocytes cells incubated during 24 h in the absence or in the presence of Aβ_25–35_ and SB203580. Values are expressed as mean ± SEM of three independent experiments, each in triplicate. * *p* < 0.05, ** *p* < 0.01, *** *p* < 0.001 *vs.* the control group.

It is known that the MAPK signaling pathway is quite important for the cellular response to external stimulation [23]. ERK1/2, p38MAPK, and JNKs are essential members of MAPK signaling pathways, which are thought to regulate the cardiac myocyte apoptosis and cardiac pathologies [24]. To further study the mechanism whereby Aβ_25–35_ induces cardiac myocyte apoptosis, we detected the phosphorylation of p38 and ERK1/2 MAPKs levels in cardiac myocytes exposed to 20 μM Aβ_25–35_. Aβ_25–35_ exposure led to a significant increase in p38 MAPK phosphorylation compared with the control group, whereas the total p38 MAPK changed little. ERK1/2 phosphorylation was decreased after Aβ_25–35_ exposure compared with the control group. The apoptosis induced by Aβ_25–35_ may be associated with an increase in the phosphor-p38 MAPK-to-total p38 MAPK ratio and a decrease in the phosphor-ERK1/2-to-total ERK1/2 ratio (Figure 5C). SB253580 as a p38 inhibitor used to detect the apoptosis status [25]. When the SB253580 was added, the apoptosis molecular markers such as cleaved caspase 3 and Bax to Bcl-2 ratio were compromised compared to Aβ_25–35_ alone (Figure 5D). At the same time we obtained a similar result in the tuned apoptosis assay which is a more accurate and sensitive assay that examines apoptosis (Figure 5E).

PD98059 is a non-ATP competitive MEK inhibitor that specifically inhibits MEK-1-mediated activation of MAPK; but does not directly inhibit ERK1 or ERK2. We used the Bliss additivity model to calculate the theoretical additivity for the combinations of 10 μM of Aβ_25–35_ and PD98059 [26]. As illustrated in Figure 5F, combinations of Aβ_25–35_ and PD98059 produced improved cell apoptosis relative to the calculated additivity, indicative of an interaction between the Aβ_25–35_ and ERK1/2 MAPKs pathway. Therefore, Aβ_25–35_ can regulate the p38 and ERK1/2 MAPKs pathway that leads to cardiac myocyte cell apoptosis.

## 3. Discussion

A novel and critical conclusion of this paper is that Aβ_25–35_ has an important toxicity toward cardiac myocytes. We have demonstrated that Aβ_25–35_ causes ER stress and affects cytoskeletal assembly, leading to the apoptosis of cardiac myocytes through activation of p38 and inhibition of Erk1/2. Through the current study reports an IC_50_ ~20 μM for cardiac effects whereas the plasma levels are low, Aβ_25–35_ could have a tiny and long term effect of a concentration-dependent removal from the brain and ultimately contribute to heart problems.

Alzheimer’s disease (AD) is the most common type of dementia. Accumulation of amyloid-beta (Aβ) peptides is considered as the most important cause associated with AD pathogenesis, which is cleaved from the amyloid precursor protein (APP) [27]. Cardiovascular risk is also prevalent and increases in the elderly AD patient [28]. By applying the genome-wide association studies (GWAS) method to AD pathogenesis it is found that cardiovascular disease contributes to AD [29]. Recently there have been more literature reports of associations between cardiovascular risk factors and AD [30,31,32,33]. Although the mechanisms for these associations are uncertain we hypothesize Aβ may affect the cardiovascular system and heart. Interestingly, our results provide clues as to the link between these diseases. Although Aβ or other amyloid precursor proteins have the ability to activate a programmed nervous cell death pathway and contribute to the pathology of the Alzheimer’s disease, our study demonstrates that Aβ_25–35_ can also trigger such cell death in cardiac myocytes isolated from rat. We believe that these finding will be very useful for future studies of AD and heart disease.

Here, using primary cultures of rat cardiac myocytes with different concentrations of Aβ_25–35_, we firstly evaluated the toxicity of Aβ_25–35_. Aβ_25–35_ could inhibit the viability of rat cardiac myocytes in a dose-dependent way, suggesting Aβ may have the same effect. The dysfunction of cellular organelles such as the endoplasmic reticulum (ER) happens in AD pathogenesis and ER stress markers have been discovered in the brain of AD patients [34]. The ER stress marker is also up-regulated in cultured neurons treated with fibrillar or oligomeric Aβ [35]. Therefore we have detected whether Aβ_25–35_ would cause the ER stress in cardiac myocytes. Under ER stress, UPR will be activated to restore ER homeostasis and then the ER stress sensor IRE-1α is activated increasing the specific splicing of XBP-1 mRNA and C/EBP homologous protein (CHOP) [36]. Subsequently, XBP-1 will up-regulate the chaperones protein GRP78 which is ER-resident [37]. After treatment of Aβ_25–35_, the GRP78 and XBP-1 as well as CHOP were up-regulated in a concentration dependent way. In addition, under prolonged ER stress conditions, apoptotic cell death is induced and is mediated either by activation of the ER associated caspase3-activation or CHOP can down-regulate the level of anti-apoptotic protein Bcl-2, further leading to apoptosis [38,39]. Following the above experiments we have examined the apoptosis status of the cardiac myocytes induced by Aβ_25–35_. In the result there is obvious apoptotic appearance from the morphology to the nucleus. Subsequently, quantitative analysis of apoptotic cells was performed. Aβ_25–35_ resulted in significant, dose-dependent induction of apoptosis evidenced by the increased apoptosis markers such as cleaved caspase-3, 7, cleaved PARP, as well as an increased ratio of Bax/Bcl-2. Meanwhile Aβ_25–35_ also affected the cytoskeletal assembly of cardiac myocytes. Nonetheless, these results are consistent with our thoughts, and we can conclude that the activation of ER stress by Aβ_25–35_ led to subsequent cellular dysfunction and apoptosis.

It is known that the MAPK signaling pathway plays an important role in cell ER stress response, apoptosis, cytoskeletal reorganization, and transcriptional regulation of genes in differentiation, proliferation, and inflammation [40]. Abundant literature has reported that MAPK was activated to reply to growth stimuli, promoting cell growth [41]. ER-mediated MAPK activation exists in the cardiovascular system [42]. In search of the mechanism underlying ER-stress and the apoptosis induced by Aβ_25–35_, we found there is an increase in p38 and a decrease in ERK phosphorylation in cardiac myocytes during exposure of Aβ_25–35_ for 24 h. The literature has reported that Aβ_25–35_ induced significant ERK activation after 5 min that gradually weakened after 6 h in a time-dependent manner in neonatal cardiomyocytes and in neonatal cardiomyocytes and in α_1A_-AR harboring CHO cells [43]. However, in our results Aβ_25–35_ inhibited ERK phosphorylation during the exposure of Aβ_25–35_ for 24 h, suggesting the length of exposure time of Aβ_25–35_ had great influence on the ERK response. Aβ_25–35_ could regulate the levels of ERK and p38 phosphorylation, suggesting that the MAPK pathway might be involve in its cardiac myocyte-protective effects. However, P38 phosphorylation may trigger cell apoptosis by differentially regulating the expression and activity of pro- and anti-apoptotic Bcl-2 family proteins. SB203580 as a selective p38 MAPK inhibitor that can inhibit the activity of p38. SB203580 exerts its inhibitory effect by binding the ATP binding pocket of p38 and has been used to identify the p38 phosphorylation in cultured cells in several previous reports [44]. P38 are a class of mitogen-activated protein kinases that are activated by a variety of cellular stresses including osmotic shock, inflammatory cytokines, lipopolysaccharides (LPS), and ultraviolet light, and are involved in adaptation to stress, apoptosis or cell differentiation [45]. The ERK1/2 could phosphorylate a number of substrates important for cell proliferation, cell cycle progression, cell division and differentiation [46]. Our results have shown that Aβ_25–35_ has different effects on p38 and ERK1/2, suggesting Aβ_25–35_-induced ER stress will result in the high p38 expression and inhibit myocyte proliferation by decreasing the ERK1/2 expression.

In our study we found that apoptosis induced by Aβ_25–35_ was prevented by SB203580 in cardiac myocytes and SB203580 abolished the level of apoptotic markers as well as the apoptotic cells by utilizing a tunel-based assay, suggesting that Aβ_25–35_ induced ER stress and apoptotic effects at least partially through modulating the MAPK pathway. However, more work is needed to prove these conclusions *in vivo* and this work is ongoing in our laboratory.

## 4. Experimental

### 4.1. Cells and Culture Conditions

Rat cardiac myocytes were isolated from the heart of young rats as described previously [9]. Briefly, the rats were anesthetized of sodium pentobarbital (150 mg/kg) and heparin (300 U/kg). After waiting until the rat is not responsive, the abdomen is sprayed with 70% EtOH, the thorax cut open and the heart removed above the aortic arch. The rat heart was excised and retrogradely perfused on a Langendorff apparatus with Ca^2+^-Tyrode’s solution(in mM NaCl 135 mM, KCl 5.4 mM, MgCl_2_ 1.0 mM, NaH_2_PO_4_ 0.33 mM, glucose 5 mM, and HEPES 10 mM, pH 7.4) via the aorta at a perfusion rate of 6 mL/min for 5 min. Then, the heart was perfused with Tyrode’s solution containing CaCl_2_ (34 mM) and collagenase II (300 mg/L) for 20 min. The temperature was at 37 °C. Finally, the heart was removed and cut into smaller pieces of 1–3 mm^3^ in PBS solution containing 0.1% tyrisin for 25 min at 37 °C. After the digestion, the cells were centrifuged for 7 min at 1500 r/min, and the pellet was resuspended. Then single myocytes were harvested after filtration through a nylon mesh (pore size 200 mm) and stored at room temperature for at least 20 min, then the supernatant were changed twice. Finally cells were placed in DMEM culture with 10% FBS without moving for 24 h, which was changed once every two days. The animal care and the experimental protocol were approved by the Animal Ethics Committee of China Medical University.

### 4.2. Reagents

Aβ_25–35_ (Y-0044, BIOSS, Beijing, China) and SB203580 (Selleck chemicals, Houston, TX, USA) were dissolved in DMSO (Sigma, St. Louis, MO, USA), and stored in aliquots at −20 °C for no more than 1 month before use. The vehicle (DMSO) was used as a control in *in vitro* experiments at a maximum concentration of 0.1%. The Hoechst dye was obtained were from the Beyotime Institute of Biothechnology (Nantong, China). *In Situ* Cell Death Detection Kit (11684817910) was bought from Roche Applied Science (Mannheim, Germany). AnnexinV/PI kit was from BD Company (Franklin, NJ, USA). Chemicals and biochemistry reagents were obtained from Sigma-Aldrich. The following antibodies were used at appropriate concentrations as recommended by the manufacturer: anti-Bax (#BA0315, Boster, wuhan, China), anti-Bcl-2 (#BA0412, Boster), cleaved Caspase-3 antibody(bs-0081R, BIOON), cleaved Caspase-3 antibody (#sc-6138, Santa Cruz, Santa Cruz, CA, USA), cleaved PARP antibody (#sc-25780, Santa Cruz), GRP78 antibody(#sc-1051, Santa Cruz), XBP-1 antibody (#sc-7160, Santa Cruz), CHOP antibody (#sc-575, Santa Cruz), Erk antibody (#sc-292838, Santa Cruz), p-ERK1/2 antibody (#4370, CST), p38 antibody (#sc-7149, Santa Cruz), p-p38 antibody (#sc-101759, Santa Cruz), p-ROCK/ROCK(#83513, Abcam, Cambridge, MA, USA), Antibodies for β-actin were purchased from LSBio (Seattle, WA, USA).

### 4.3. Viability Assay

Viability studies were performed using 3-(4, 5-dimethylthiaz-ol-2-yl)-2, 5-diphenyltetrazoliumbromide (MTT, Sigma) as described previously [47]. After cells were incubated in 96-well plates (100 μL) in the presence or absence of Aβ_25–35_ for 24 h, MTT was added and the plate was incubated for 4–6 h at 37 °C. The absorbance (A) was measured at 570 nm and survival ratio (%) was calculated using the following equation: survival ratio (%) = (A_treatment_/A_control_) × 100%. IC_50_ was taken as the concentration that caused 50% inhibition of cell viabilities and calculated by the Graphpad Prism 5 software.

### 4.4. Cell Morphology Assessment

To detect morphological evidence of apoptosis induced by Aβ_25–35_, rat cardiac myocytes cell nuclei were visualized following DNA staining with the fluorescent dye DAPI. Briefly, cells were seeded in 6-well culture plates and treated with indicated concentration of Aβ_25–35_. At the end of incubation, the morphology of cells was monitored under an inverted light microscope. Cells were then fixed with 4% paraformaldehyde for 20 min and washed with PBS, and incubated with Hoechst dye (1 mg/mL) for 10 min. After washing with PBS, cells were observed under a fluorescence microscope (Olympus, Thornwood, NY, USA).

### 4.5. TUNEL-Based Assay

TUNEL staining was performed using an *In Situ* Cell Death Detection Kit, Fluorescein (Roche Applied Science), according to the manufacturer’s directions. Cells were fixed in 4% paraformaldehyde for 15 min at room temperature, then washed three times in PBS for 5 min each. Then, slides were were rinsed twice in PBS buffer and immersed in 0.1% (v/v) Triton X-100 supplemented with 0.1% (w/v) sodium citrate in ice-cold PBS, for 2 min, to permeabilize the cells. Slides were rinsed again three times with PBS and incubated with TUNEL mixture for 1 h at 37 °C, in the dark. Finally, slides were rinsed three times with PBS and mounted with Dako Cytomation Fluorescent solution (Dako Cytomation, Carpinteria, CA, USA) onto a microscope slide for visualization in fluorescence microscope (Olympus).

### 4.6. Apoptosis Assay

Rat cardiac myocytes (2 × 10^5^) were seeded on 60 mm culture dishes in culture medium containing 10% FBS. The following day the cells were treated with the indicated concentrations of Aβ_25–35_ for 48 h. After treatment, all cells, including both floating and attached cells, were collected. According to the supplier’s protocol the apoptotic cells were detected with an Annexin V-FITC Apoptosis Detection Kit (BD Biosciences, San Diego, CA, USA) by using a FacScan analyzer (argon laser; Becton, Dickinson and Company (BD), USA). All data were analyzed using the Flow software.

### 4.7. Immunofluorescence Assays

After the treatment, rat cardiac myocytes in coverslips were washed three times with ice-cold PBS and fixed with freshly prepared 4% paraformaldehyde in PBS for 15 min. After the fixation, cells were soaked with PBS three times for 5 min and then permeabilized with 0.5% Triton X-100 in PBS for 30 min at room temperature. Subsequently, the cells were again washed with PBS twice for 5 min and blocked with 1% phalloidin for 60 min at room temperature in the dark. Then the phalloidin were washed and cells were incubated with DAPI. Finally the DAPI were washed and a drop of anti-fluorescence quencher was added. To visualize actin filaments, the treated cells were directly labeled with phalloidin conjugated with Alexa Fluor 488 and analyzed under a fluorescence microscope after counterstaining with DAPI.

### 4.8. Immunoblotting

Rat cardiac myocytes were pretreated with various concentrations of Aβ_25–35_. After the stimulation, cells were collected and lysed in NP-40 lysis buffer (50 mmol/L Tris-HCl, 1% NP40, 40 mmol/L NaF, 150 mmol/L NaCl, 10 μmol/L/mL Na_3_VO_4_/phenylmethylsulfonyl fluoride/DTT; and 1 mg/mL leupeptin, aprotinin, and trypsin inhibitor), boiled for 10 min, followed by brief sonication. Lysates were then cleared by centrifugation at 14,000 × g for 10 min, and the supernatant was collected. Protein concentration was determined using the BCA kit (Pierce Chemical, Rockford, IL, USA) as per the manufacturer’s instructions. 50 or 100 μg of protein were resolved by SDS-PAGE and transferred onto nitrocellulose membranes. Blots were incubated overnight at 4 °C with primary antibodies followed by horseradish peroxidase–conjugated secondary antibodies for 2 h. Detection was performed by chemiluminescence (ECL, Qihai technology, Shanghai, China). All blots were stripped and incubated with polyclonal anti-β-actin antibody to ascertain equal loading of proteins.

### 4.9. Statistical Analysis

Nonlinear mixed models were used to obtain IC_50_. All data represent at least three independent experiments and are displayed as the mean ± SEM. Two-tailed student’s *t*-test was used to evaluate statistical significance of difference between treated and control groups.

## 5. Conclusions

In summary, our data suggest that Aβ_25–35_ plays a critical role by causing the ER stress and apoptosis mediated by p38 and ERK1/2 phosphorylation in cardiac myocyte cells isolated from rat. Because the relationship between cardiac cardiovascular disease and AD is unclear, our finding represents a major step forward toward understanding the molecular mechanisms underlying Aβ and could contribute to the study of cardiovascular and AD disease as well as treatment strategies for these two groups of patients.

## References

[1] Ballard C., Gauthier S., Corbett A., Brayne C., Aarsland D., Jones E. (2011). Alzheimer’s disease. Lancet.

[2] Jack C.R., Knopman D.S., Jagust W.J., Petersen R.C., Weiner M.W., Aisen P.S., Shaw L.M., Vemuri P., Wiste H.J., Weigand S.D. (2013). Tracking pathophysiological processes in Alzheimer’s disease: An updated hypothetical model of dynamic biomarkers. Lancet Neurol..

[3] Zheng H., Koo E.H. (2011). Biology and pathophysiology of the amyloid precursor protein. Mol. Neurodegener..

[4] O’Brien R.J., Wong P.C. (2011). Amyloid precursor protein processing and Alzheimer’s disease. Annu. Rev. Neurosci..

[5] Knauer M.F., Soreghan B., Burdick D., Kosmoski J., Glabe C.G. (1992). Intracellular accumulation and resistance to degradation of the Alzheimer amyloid a4/beta protein. Proc. Natl. Acad. Sci. USA.

[6] Pike C.J., Walencewicz-Wasserman A.J., Kosmoski J., Cribbs D.H., Glabe C.G., Cotman C.W. (1995). Structure-activity analyses of beta-amyloid peptides: Contributions of the beta 25–35 region to aggregation and neurotoxicity. J. Neurochem..

[7] Halverson K., Fraser P.E., Kirschner D.A., Lansbury P.T. (1990). Molecular determinants of amyloid deposition in Alzheimer’s disease: Conformational studies of synthetic beta-protein fragments. Biochemistry.

[8] Shanmugam G., Polavarapu P.L. (2004). Structure of A*β*(25–35) peptide in different environments. Biophys. J..

[9] Turdi S., Guo R., Huff A.F., Wolf E.M., Culver B., Ren J. (2009). Cardiomyocyte contractile dysfunction in the APPswe/PS1dE9 mouse model of Alzheimer’s disease. PLoS One.

[10] Fidzianska A., Walczak E., Bekta P., Chojnowska L. (2011). Are cardiomyocytes able to generate pre-amyloid peptides?. Folia Neuropathol..

[11] Chen W., Dilsizian V. (2012). Molecular imaging of amyloidosis: Will the heart be the next target after the brain?. Curr. Cardiol. Rep..

[12] Yan J., Young M.E., Cui L., Lopaschuk G.D., Liao R., Tian R. (2009). Increased glucose uptake and oxidation in mouse hearts prevent high fatty acid oxidation but cause cardiac dysfunction in diet-induced obesity. Circulation.

[13] Wang S., Zhang C., Sheng X., Zhang X., Wang B., Zhang G. (2014). Peripheral expression of mapk pathways in Alzheimer’s and Parkinson’s diseases. J. Clin. Neurosci..

[14] Davis J., Cribbs D.H., Cotman C.W., van Nostrand W.E. (1999). Pathogenic amyloid beta-protein induces apoptosis in cultured human cerebrovascular smooth muscle cells. Amyloid.

[15] Tong L., Thornton P.L., Balazs R., Cotman C.W. (2001). Beta-amyloid-(1-42) impairs activity-dependent camp-response element-binding protein signaling in neurons at concentrations in which cell survival is not compromised. J. Biol. Chem..

[16] Bashir M., Parray A.A., Baba R.A., Bhat H.F., Bhat S.S., Mushtaq U., Andrabi K.I., Khanday F.A. (2014). Beta-amyloid-evoked apoptotic cell death is mediated through MKK6-p66SHC pathway. Neuromol. Med..

[17] Yankner B.A., Dawes L.R., Fisher S., Villa-Komaroff L., Oster-Granite M.L., Neve R.L. (1989). Neurotoxicity of a fragment of the amyloid precursor associated with Alzheimer’s disease. Science.

[18] Costa R.O., Ferreiro E., Oliveira C.R., Pereira C.M. (2013). Inhibition of mitochondrial cytochrome C oxidase potentiates abeta-induced er stress and cell death in cortical neurons. Mol. Cell. Neurosci..

[19] Lee A.H., Iwakoshi N.N., Glimcher L.H. (2003). Xbp-1 regulates a subset of endoplasmic reticulum resident chaperone genes in the unfolded protein response. Mol. Cell. Biol..

[20] Oyadomari S., Mori M. (2004). Roles of CHOP/GADD153 in endoplasmic reticulum stress. Cell Death Differ..

[21] Ward M.W., Concannon C.G., Whyte J., Walsh C.M., Corley B., Prehn J.H. (2010). The amyloid precursor protein intracellular domain(aicd) disrupts actin dynamics and mitochondrial bioenergetics. J. Neurochem..

[22] Riento K., Ridley A.J. (2003). Rocks: Multifunctional kinases in cell behaviour. Nat. Rev. Mol. Cell Biol..

[23] Kim E.K., Choi E.J. (2010). Pathological roles of mapk signaling pathways in human diseases. Biochim. Biophys. Acta.

[24] Li D.Y., Tao L., Liu H., Christopher T.A., Lopez B.L., Ma X.L. (2006). Role of ERK1/2 in the anti-apoptotic and cardioprotective effects of nitric oxide after myocardial ischemia and reperfusion. Apoptosis.

[25] Jones E.V., Dickman M.J., Whitmarsh A.J. (2007). Regulation of p73-mediated apoptosis by c-Jun N-terminal kinase. Biochem. J..

[26] Goldoni M., Johansson C. (2007). A mathematical approach to study combined effects of toxicants *in vitro*: Evaluation of the bliss independence criterion and the loewe additivity model. Toxicol. In Vitro.

[27] Ittner L.M., Gotz J. (2011). Amyloid-beta and tau—A toxic pas de deux in Alzheimer’s disease. Nat. Rev. Neurosci..

[28] Rees K., Dyakova M., Wilson N., Ward K., Thorogood M., Brunner E. (2013). Dietary advice for reducing cardiovascular risk. Cochrane Database Syst. Rev..

[29] Liu G., Yao L., Liu J., Jiang Y., Ma G., Chen Z., Zhao B., Li K. (2014). Cardiovascular disease contributes to Alzheimer’s disease: Evidence from large-scale genome-wide association studies. Neurobiol. Aging.

[30] Martins I.J., Hone E., Foster J.K., Sunram-Lea S.I., Gnjec A., Fuller S.J., Nolan D., Gandy S.E., Martins R.N. (2006). Apolipoprotein E, cholesterol metabolism, diabetes, and the convergence of risk factors for Alzheimer’s disease and cardiovascular disease. Mol. Psychiatry.

[31] Van Kan A.G., Rolland Y., Nourhashemi F., Coley N., Andrieu S., Vellas B. (2009). Cardiovascular disease risk factors and progression of Alzheimer’s disease. Dement. Geriatr. Cogn. Disord..

[32] Rosendorff C., Beeri M.S., Silverman J.M. (2007). Cardiovascular risk factors for Alzheimer’s disease. Am. J. Geriatr. Cardiol..

[33] Epstein N.U., Xie H., Ruland S.D., Pandey D.K. (2012). Vascular risk factors and cardiovascular outcomes in the Alzheimer’s disease neuroimaging initiative. Am. J. Alzheimer’s Dis. Other Dement..

[34] Hoozemans J.J., Veerhuis R., van Haastert E.S., Rozemuller J.M., Baas F., Eikelenboom P., Scheper W. (2005). The unfolded protein response is activated in Alzheimer’s disease. Acta Neuropathol..

[35] Resende R., Ferreiro E., Pereira C., Oliveira C.R. (2008). ER stress is involved in abeta-induced Gsk-3beta activation and TAU phosphorylation. J. Neurosci. Res..

[36] Dalton L.E., Clarke H.J., Knight J., Lawson M.H., Wason J., Lomas D.A., Howat W.J., Rintoul R.C., Rassl D.M., Marciniak S.J. (2013). The endoplasmic reticulum stress marker CHOP predicts survival in malignant mesothelioma. Br. J. Cancer.

[37] Pfaffenbach K.T., Lee A.S. (2011). The critical role of GRP78 in physiologic and pathologic stress. Curr. Opin. Cell Biol..

[38] Scull C.M., Tabas I. (2011). Mechanisms of er stress-induced apoptosis in atherosclerosis. Arterioscler. Thromb. Vasc. Biol..

[39] Verfaillie T., Garg A.D., Agostinis P. (2013). Targeting er stress induced apoptosis and inflammation in cancer. Cancer Lett..

[40] Shi J., Guan J., Jiang B., Brenner D.A., del Monte F., Ward J.E., Connors L.H., Sawyer D.B., Semigran M.J., Macgillivray T.E. (2010). Amyloidogenic light chains induce cardiomyocyte contractile dysfunction and apoptosis via a non-canonical p38alpha MAPK pathway. Proc. Natl. Acad. Sci. USA.

[41] Cargnello M., Roux P.P. (2011). Activation and function of the MAPKs and their substrates, the MAPK-activated protein kinases. Microbiol. Mol. Biol. Rev..

[42] Padilla J., Jenkins N.T. (2013). Induction of endoplasmic reticulum stress impairs insulin-stimulated vasomotor relaxation in rat aortic rings: Role of Endothelin-1. J. Physiol. Pharmacol..

[43] Haase N., Herse F., Spallek B., Haase H., Morano I., Qadri F., Szijarto I.A., Rohm I., Yilmaz A., Warrington J.P. (2013). Amyloid-beta peptides activate alpha1-adrenergic cardiovascular receptors. Hypertension.

[44] Cuenda A., Rouse J., Doza Y.N., Meier R., Cohen P., Gallagher T.F., Young P.R., Lee J.C. (1995). Sb 203580 is a specific inhibitor of a map kinase homologue which is stimulated by cellular stresses and interleukin-1. FEBS Lett..

[45] Goldstein D.M., Gabriel T. (2005). Pathway to the clinic: Inhibition of p38 MAP kinase. A review of ten chemotypes selected for development. Curr. Top. Med. Chem..

[46] Pearson G., Robinson F., Beers Gibson T., Xu B.E., Karandikar M., Berman K., Cobb M.H. (2001). Mitogen-activated protein (MAP) kinase pathways: Regulation and physiological functions. Endocr. Rev..

[47] Verma A., Prasad K.N., Singh A.K., Nyati K.K., Gupta R.K., Paliwal V.K. (2010). Evaluation of the MTT lymphocyte proliferation assay for the diagnosis of neurocysticercosis. J. Microbiol. Methods.

